# Breaking Boundaries: An Unusual Case of Rectal Signet Ring Cell Carcinoma Invading the Uterus and Mesentery

**DOI:** 10.7759/cureus.83405

**Published:** 2025-05-03

**Authors:** Mitali Singh, Vaibhav Mane

**Affiliations:** 1 Pathology, Bharati Vidyapeeth (Deemed to be University) Medical College and Hospital, Sangli, IND

**Keywords:** acute anal fissure, carcinoembryonic antigen (cea), carcinoma rectum, occult blood, signet-ring cell carcinoma

## Abstract

Signet ring cell carcinoma (SRCC) of the rectum is an infrequent and highly malignant subtype of colorectal cancer. Representing a small fraction of all colorectal carcinomas, SRCC is characterised by its aggressive clinical course, delayed presentation, and poor prognosis. The diagnosis is often established at advanced stages due to its insidious onset and nonspecific symptoms. We present a rare case of a middle-aged female with primary rectal SRCC who initially presented with symptoms suggestive of anaemia and urinary abnormalities. Laboratory investigations revealed microcytic anaemia, positive stool occult blood, and significant proteinuria. Radiological imaging and histopathological examination confirmed a diagnosis of poorly differentiated signet ring cell adenocarcinoma of the rectum, with extensive metastatic spread to the uterus, mesentery, liver, and para-aortic lymph nodes. This case underscores the diagnostic complexity of rectal SRCC due to its atypical presentation and rapid progression. It emphasises the necessity for a high index of clinical suspicion, even in the absence of overt gastrointestinal symptoms, and advocates for a comprehensive diagnostic workup including advanced imaging and histological evaluation to facilitate early detection and timely intervention.

## Introduction

Signet ring cell carcinoma (SRCC) is a rare and particularly aggressive histologic subtype of colorectal adenocarcinoma, representing less than 1% of all colorectal malignancies. It is characterised by the presence of malignant epithelial cells with abundant intracellular mucin, which displaces the nucleus eccentrically, giving the classic "signet ring" appearance [1-3]. This distinct morphology is associated with a significantly poorer prognosis compared to conventional colorectal adenocarcinomas, largely due to its aggressive biological behavior.

While SRCC is most commonly encountered in the stomach, its occurrence in the rectum is exceedingly uncommon. When it does arise in the rectum, it is often diagnosed at a more advanced stage [1]. This delay in detection is attributed to its subtle and nonspecific clinical presentation, infiltrative growth pattern, and tendency to evade early endoscopic identification. Furthermore, SRCC exhibits a strong propensity for peritoneal seeding, lymphatic dissemination, and distant metastases, frequently complicating its clinical course [3].

Given its rarity and the diagnostic challenges it presents, each case of rectal SRCC warrants detailed documentation to expand the current understanding of its clinical manifestations and progression. The case presented herein underscores the aggressive nature of rectal SRCC and is further distinguished by an unusual metastatic pattern involving the uterus and mesentery - sites that are seldom reported in the literature. Such atypical presentations reinforce the importance of heightened clinical vigilance and contribute valuable insights toward improving diagnostic strategies and therapeutic approaches for this rare malignancy.

## Case presentation

A 64-year-old female presented with complaints of per rectal bleeding for one month, which had worsened over the past two weeks. The bleeding typically occurred post-defecation, accompanied by a burning sensation, though not associated with hard stools. She also reported altered bowel habits, generalised weakness, and unintentional weight loss over the preceding weeks.

Her medical history was significant for ischaemic heart disease with single-vessel involvement, for which she had recently undergone percutaneous transluminal coronary angioplasty (PTCA). She was also a known case of hypertension.

On physical examination, the patient appeared pale. Laboratory investigations revealed anaemia, with a haemoglobin level of 9.8 g/dL. Other parameters, including total leukocyte and platelet counts, renal and liver function tests, serum electrolytes, coagulation profile, and random blood glucose, were within normal limits. Urinalysis demonstrated significant proteinuria (+++), while stool examination was positive for occult blood, with numerous red blood cells and 6-7 pus cells on microscopy.

A two-dimensional (2D) echocardiographic study showed regional wall motion abnormalities in the anterior wall, anteroseptal segment, and apex, along with degenerative valvular changes and mild mitral regurgitation. Abdominal ultrasonography was inconclusive.

Upper gastrointestinal endoscopy showed evidence of antral gastritis. Colonoscopic evaluation demonstrated an ulceroproliferative lesion in the rectosigmoid region. For illustrative purposes, the colonoscopy images can be found in the appendix of this report (Appendix).

A contrast-enhanced CT scan of the abdomen and pelvis demonstrated neoplastic thickening of the rectosigmoid colon, with associated regional lymphadenopathy. Based on clinical findings and imaging, a provisional diagnosis of rectal growth with associated anal fissure, hypertension, and ischaemic heart disease (post-PTCA) was made. Serum carcinoembryonic antigen (CEA) was markedly elevated at 267 ng/mL, raising a strong suspicion of malignancy. A biopsy was obtained from the rectal lesion for definitive histopathological analysis.

**Figure 1:**
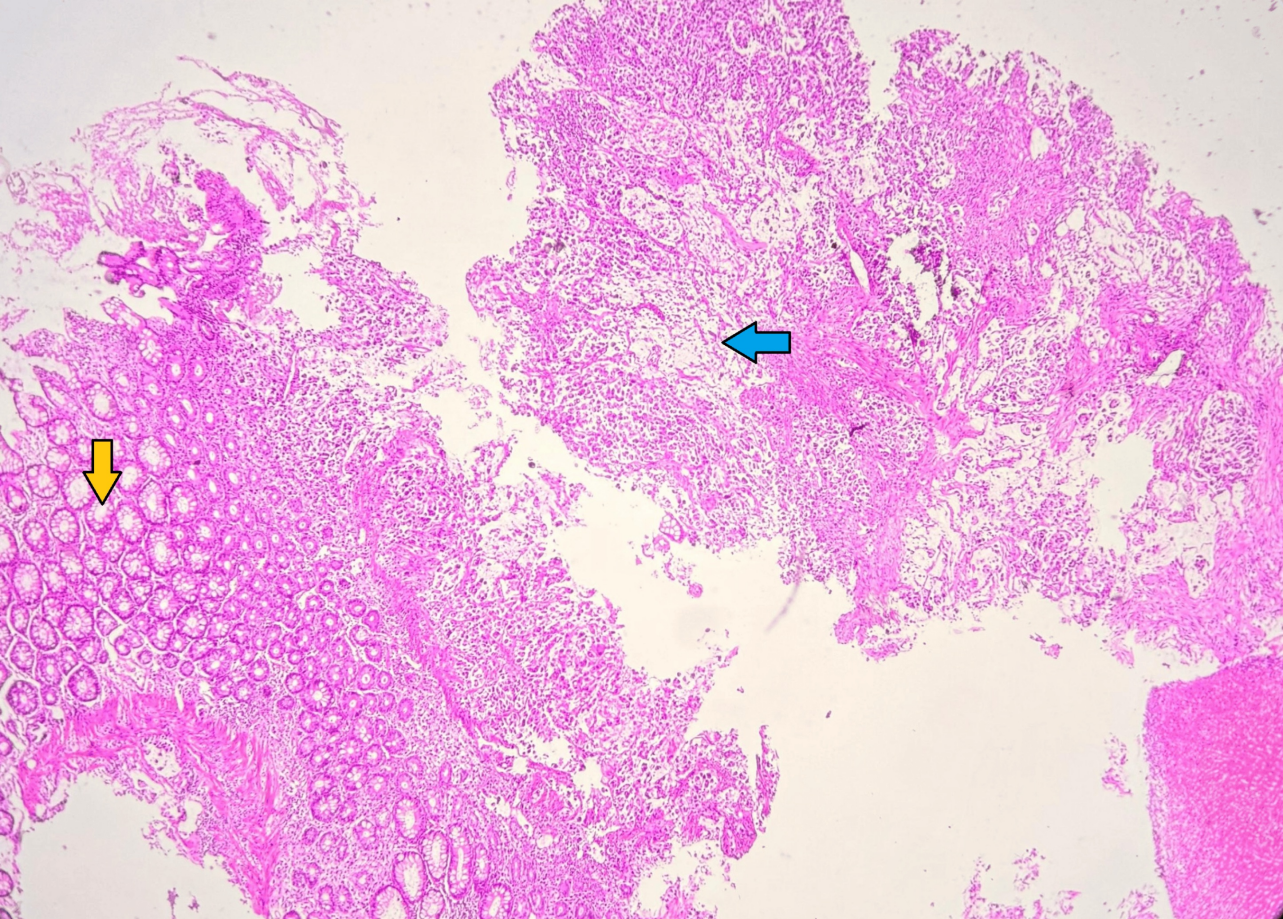
Microscopic image of H&E-stained section showing fragmented bits of rectal mucosa (yellow arrow) with a tumour (blue arrow) at 2x magnification.

**Figure 2:**
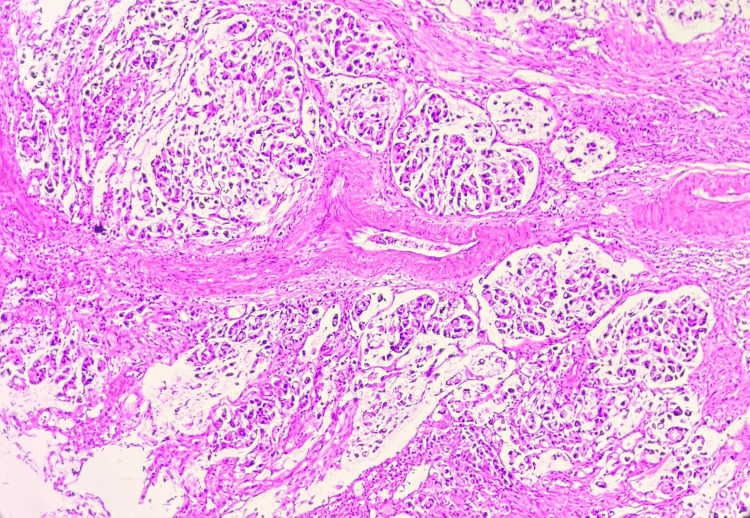
Microscopic image of H&E-stained section showing tumour cells arranged in sheets and clusters at 10x magnification.

**Figure 3:**
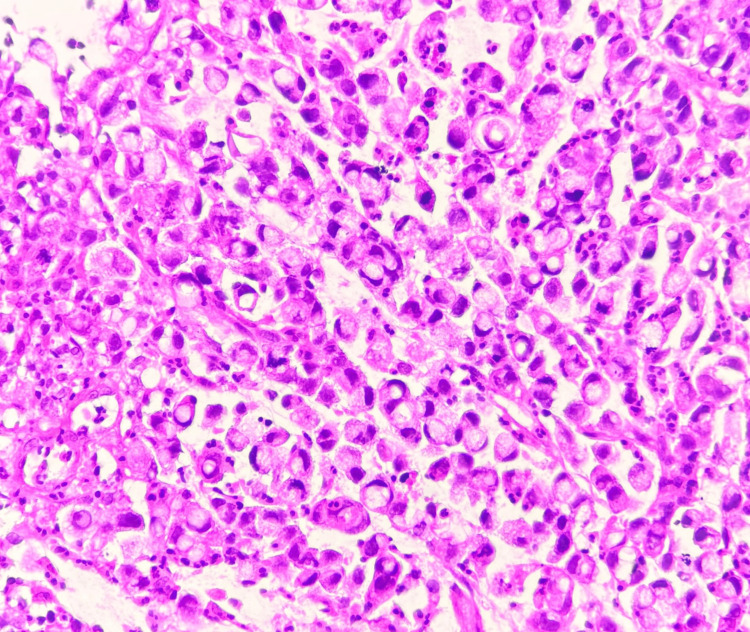
Microscopic image of H&E-stained section with round to oval tumour cells with hyperchromatic nuclei, eosinophilic cytoplasm, and displaying characteristic intracytoplasmic mucin vacuoles displacing the nuclei peripherally (crescent-shaped), forming the classic signet ring appearance at 40x magnification.

Microscopic examination of the biopsy specimen revealed features consistent with SRCC (Figure 1). The tumour was composed of round to oval cells with hyperchromatic nuclei and eosinophilic cytoplasm arranged in sheets and clusters (Figure 2). The cells displayed characteristic intracytoplasmic mucin vacuoles displacing the nuclei peripherally (crescent-shaped), forming the classic signet ring appearance. A desmoplastic stromal reaction and scattered atypical mitoses were also noted (Figure 3).

Further evaluation showed hypoalbuminaemia (serum albumin: 2.6 g/dL; total protein: 5.0 g/dL), a mild elevation of creatine kinase-MB (CK-MB) (32 IU/L), and elevated D-dimer levels (1.4 µg/mL), while troponin I remained within normal limits. Thoracic ultrasonography revealed mild bilateral pleural effusions with patchy basal consolidations.

**Figure 4:**
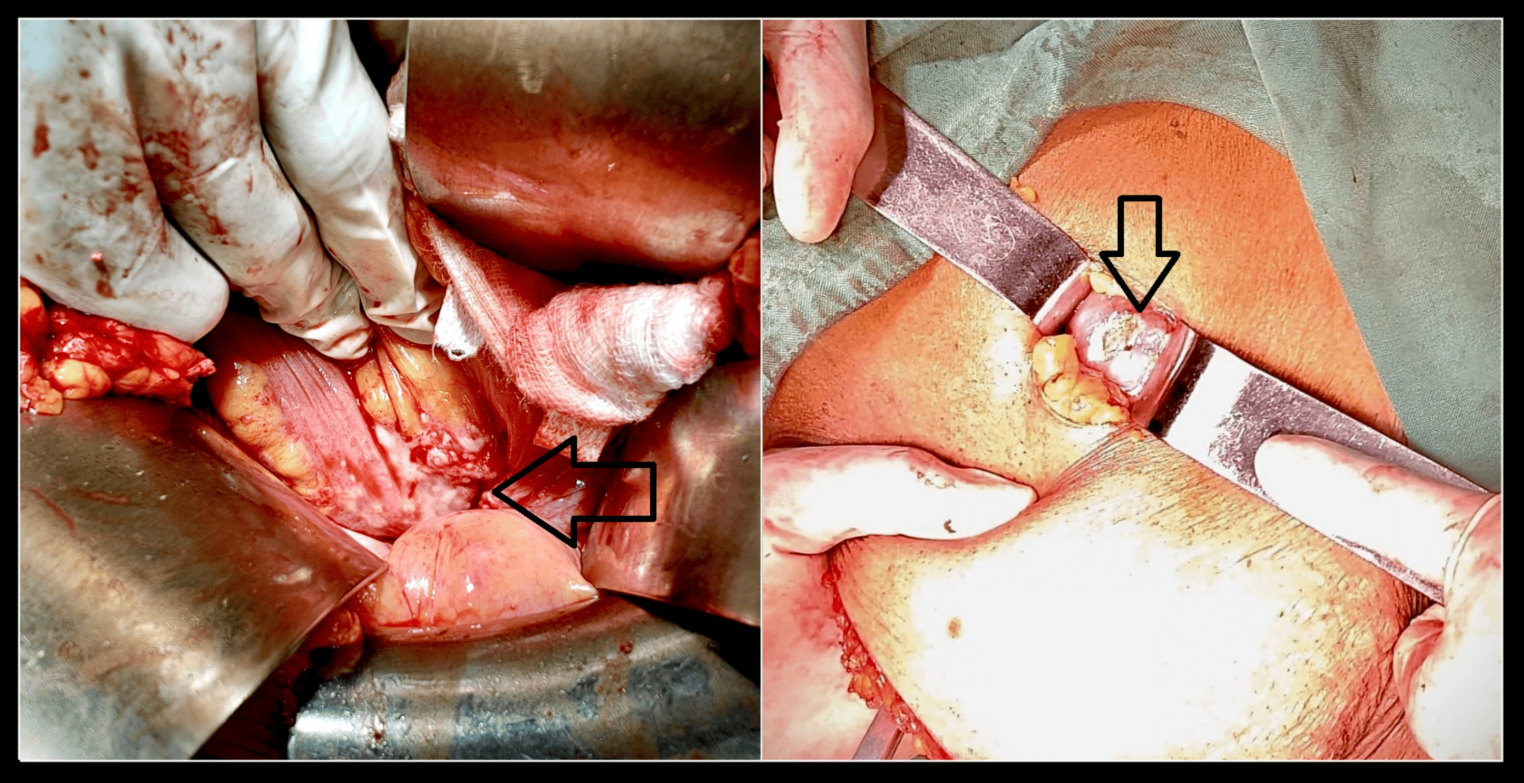
Images showing metastatic deposits of the tumour at the omentum during surgical exploration.

Given the suspicion of metastatic spread, surgical exploration was performed, which revealed multiple peritoneal deposits involving the uterine serosa, omentum, and mesentery (Figure 4).

**Figure 5:**
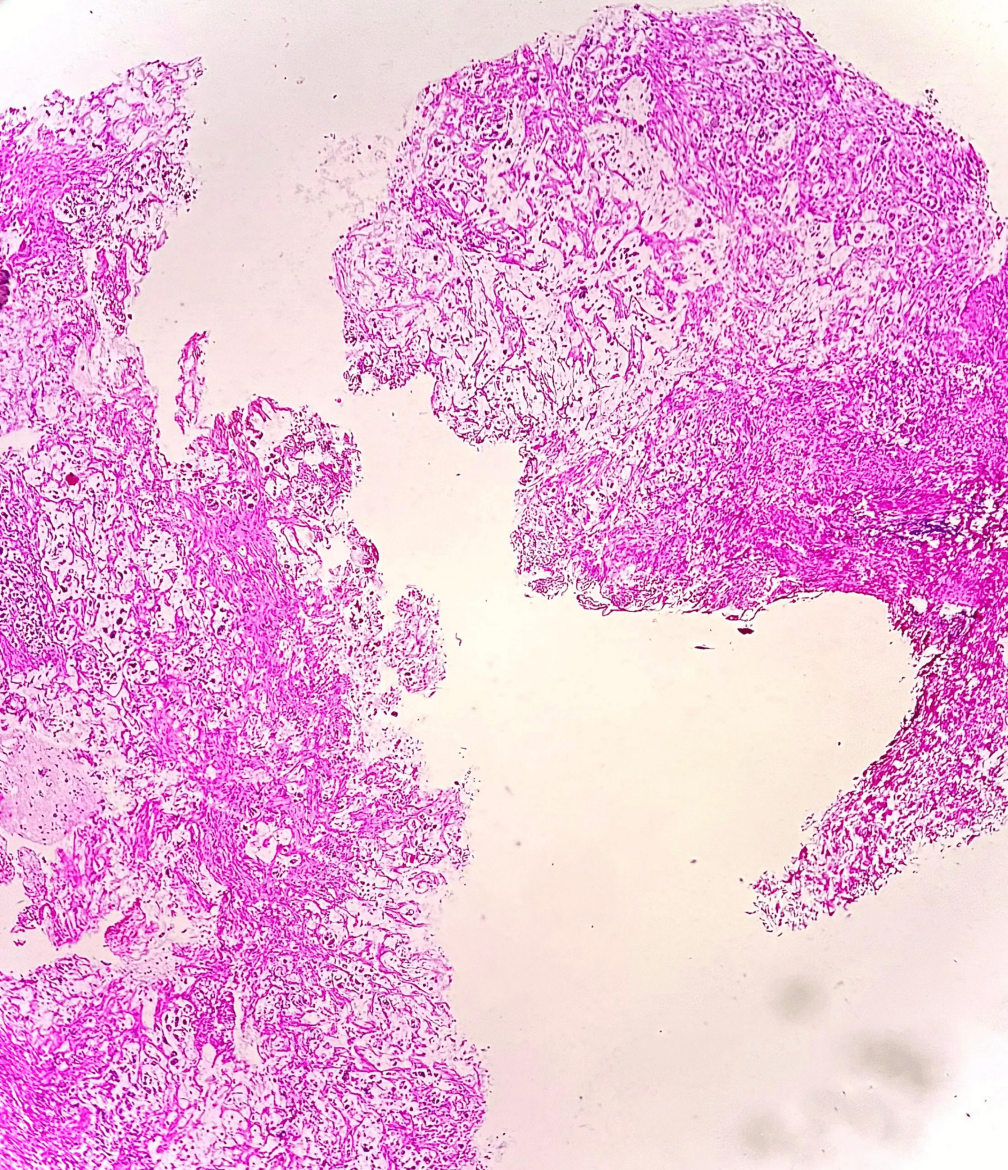
Microscopic image of H&E-stained sections showing metastatic tumour cells in the uterine wall (2x magnification).

**Figure 6:**
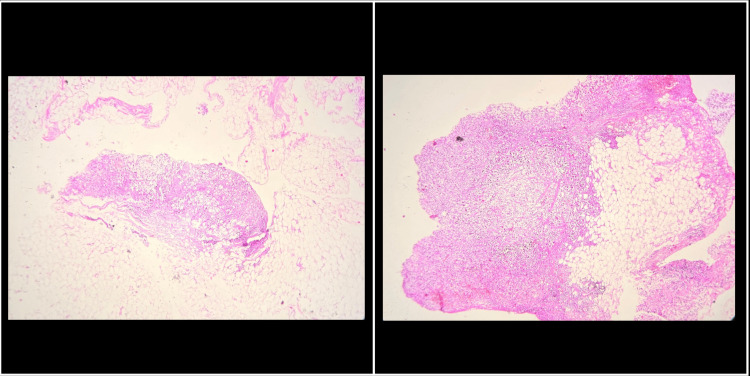
Microscopic images of H&E-stained sections showing metastatic tumour cells in the omentum (2x magnification).

Representative tissue samples from these sites were obtained and sent for histopathological examination. Microscopy confirmed the presence of metastatic SRCC, consistent with peritoneal dissemination from the primary rectal tumour (Figures 5, 6).

The patient has been started on systemic chemotherapy and remains under close multidisciplinary follow-up. As the case is recent, treatment response and long-term outcomes are yet to be determined.

## Discussion

Rectal SRCC is a rare and aggressive histologic subtype of colorectal cancer, comprising less than 1% of all colorectal malignancies [1-3]. Its distinctive morphology - characterised by malignant epithelial cells containing abundant intracellular mucin that displaces the nucleus to the cell periphery - contributes to its classification and aggressive clinical course [4].

Unlike conventional colorectal adenocarcinomas that often present with changes in bowel habits or signs of luminal obstruction [5], SRCC tends to grow in a diffuse infiltrative pattern. This allows it to spread extensively within the bowel wall without forming discrete masses, often delaying clinical detection. As a result, patients may present with nonspecific symptoms such as fatigue, iron-deficiency anaemia, and intermittent rectal bleeding, as was observed in this case. The absence of hallmark obstructive signs frequently leads to misdirection during the diagnostic process [4]. In this patient, the presence of significant proteinuria and a lack of definitive gastrointestinal symptoms initially raised concerns for renal pathology, contributing to a delay in establishing the primary diagnosis.

This case also illustrates an uncommon metastatic pattern for rectal SRCC, with tumour infiltration involving the uterus and mesentery. These findings were not evident on initial imaging and only became apparent upon further evaluation following new systemic signs, such as elevated cardiac enzymes and a newly detected cardiac murmur. Repeat imaging demonstrated abnormal soft-tissue densities in the mesentery and uterus, and subsequent biopsy confirmed metastatic SRCC in these sites.

SRCC is known for its aggressive behaviour, including early peritoneal dissemination [4] and a tendency to spread transmurally to adjacent organs. In some cases, extensive metastatic spread may occur even in the absence of overt lymphadenopathy, as was seen here. The tumour’s infiltrative nature allows it to cross tissue planes and serosal surfaces, bypassing traditional lymphovascular routes of metastasis [6]. This capacity for silent dissemination contributes to the advanced stage at which most cases are diagnosed. Notably, peritoneal carcinomatosis is a common pattern of failure in SRCC, often leading to rapid disease progression and poor prognosis [7].​

Therapeutically, SRCC carries a poor prognosis due to both delayed diagnosis and resistance to standard chemotherapy regimens [8]. Its mucin-rich cellular composition and diffuse growth pattern limit responsiveness to treatment, and surgical options are often constrained by the extent of metastatic involvement at presentation. Emerging evidence suggests that adjuvant chemotherapy may offer survival benefits in certain cases, particularly when initiated early in the disease course [9].​

This case emphasises the importance of maintaining a high index of suspicion when evaluating patients with unexplained anaemia and positive faecal occult blood tests, even in the absence of classical gastrointestinal symptoms. Early colonoscopy and comprehensive imaging are essential to detect subtle but clinically significant pathology. Additionally, unusual sites of metastasis should be considered in aggressive colorectal subtypes like SRCC [6], as early recognition of disseminated disease may inform both prognosis and management decisions. Clinicians should also be aware of atypical metastatic presentations, such as involvement of the bone marrow and skin, which have been documented in SRCC cases [10,11].

## Conclusions

This case exemplifies the diagnostic challenges associated with rectal SRCC, particularly when presenting with atypical symptoms and an unusual pattern of metastasis. The involvement of the uterus and mesentery in this patient underscores the aggressive nature of SRCC and its ability to invade contiguous structures, often in the absence of classic signs of advanced colorectal cancer. This highlights the need for comprehensive imaging, prompt biopsy, and histological evaluation in patients with persistent, unexplained gastrointestinal and systemic symptoms.

Early recognition of this rare tumour subtype is imperative, as delayed diagnosis often results in extensive metastatic disease and limited therapeutic options. A multidisciplinary approach involving oncologists, radiologists, gastroenterologists, and pathologists is essential for optimising diagnostic accuracy and tailoring individualised treatment plans. Future research into targeted therapies and early diagnostic biomarkers for SRCC may offer improved outcomes in such challenging cases.

## Figures and Tables

**Figure 7:**
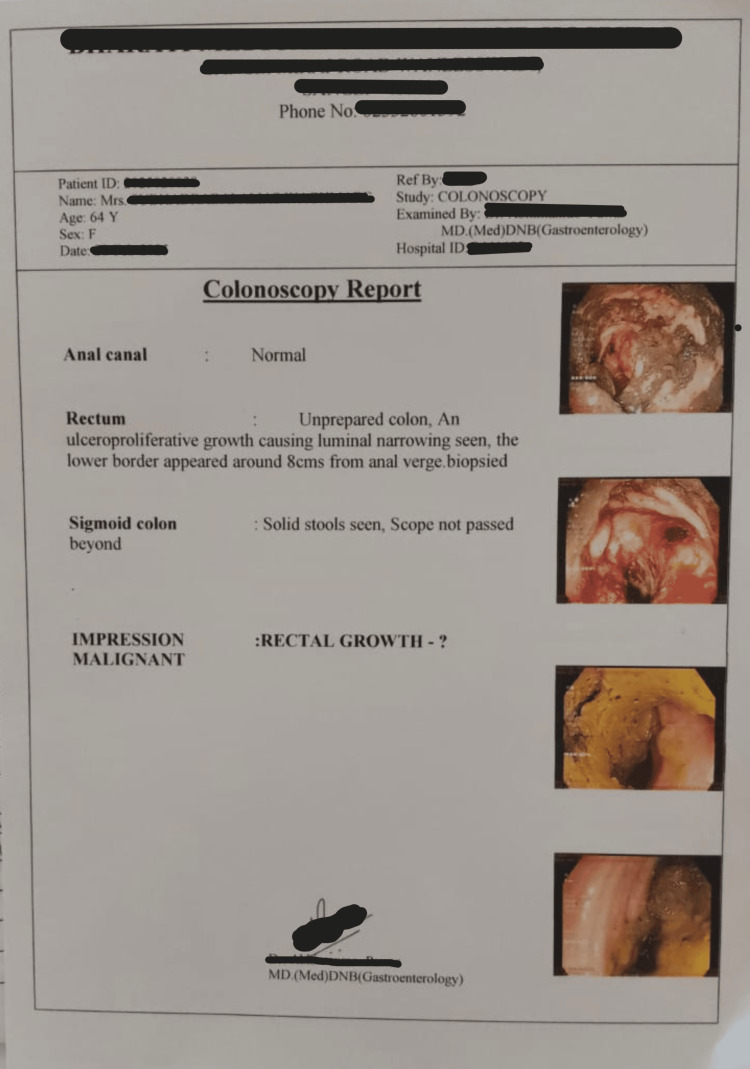
Colonoscopy report of the patient showing normal anal canal, rectum with an ulceroproliferative growth causing luminal narrowing and solid stools at the sigmoid colon (scope not passed beyond).
